# Supplementary material for: Transcriptome Profiling of Phenylalanine-Treated Human Neuronal Model: Spotlight on Neurite Impairment and Synaptic Connectivity
Source: Int J Mol Sci. 2024 Sep 18;25(18):10019. doi: 10.3390/ijms251810019 (PMC11431966; doi:10.3390/ijms251810019)
Supplement: Supplementary file 1 [file ijms-25-10019-s001.zip › Stankovic_et_al_Supplementary_figures.pdf]

SUPPLEMENTARY MATERIALS

Transcriptome Profiling of Phenylalanine-Treated Human Neuronal Model: Spotlight on Neurite Impairment and Synaptic Connectivity

Sara Stankovic 1, Andrijana Lazic 1, Marina Parezanovic 1, Milena Stevanovic 1,2,3, Sonja Pavlovic 1,

Maja Stojiljkovic 1 and Kristel Klaassen 1,*

1 Institute of Molecular Genetics and Genetic Engineering, University of Belgrade, Vojvode Stepe 444a, 11042 Belgrade, Serbia; sstankovic@imgge.bg.ac.rs (S.S.); andrijanak@imgge.bg.ac.rs (A.L.); marina.parezanovic@imgge.bg.ac.rs (M.P.); milenastevanovic@imgge.bg.ac.rs (M.S.); sonya@imgge.bg.ac.rs (S.P.); maja.stojiljkovic@imgge.bg.ac.rs (M.S.)
2 Institute of Physiology and Biochemistry "Ivan Djaja", Faculty of Biology, University of Belgrade, Studentski trg 16, 11158 Belgrade, Serbia
3 Serbian Academy of Sciences and Arts, Kneza Mihaila 35, 11001 Belgrade, Serbia
* Correspondence: kristel.klaassen@imgge.bg.ac.rs; Tel.: +381-11-3976-445; Fax: +381-11-3975-808

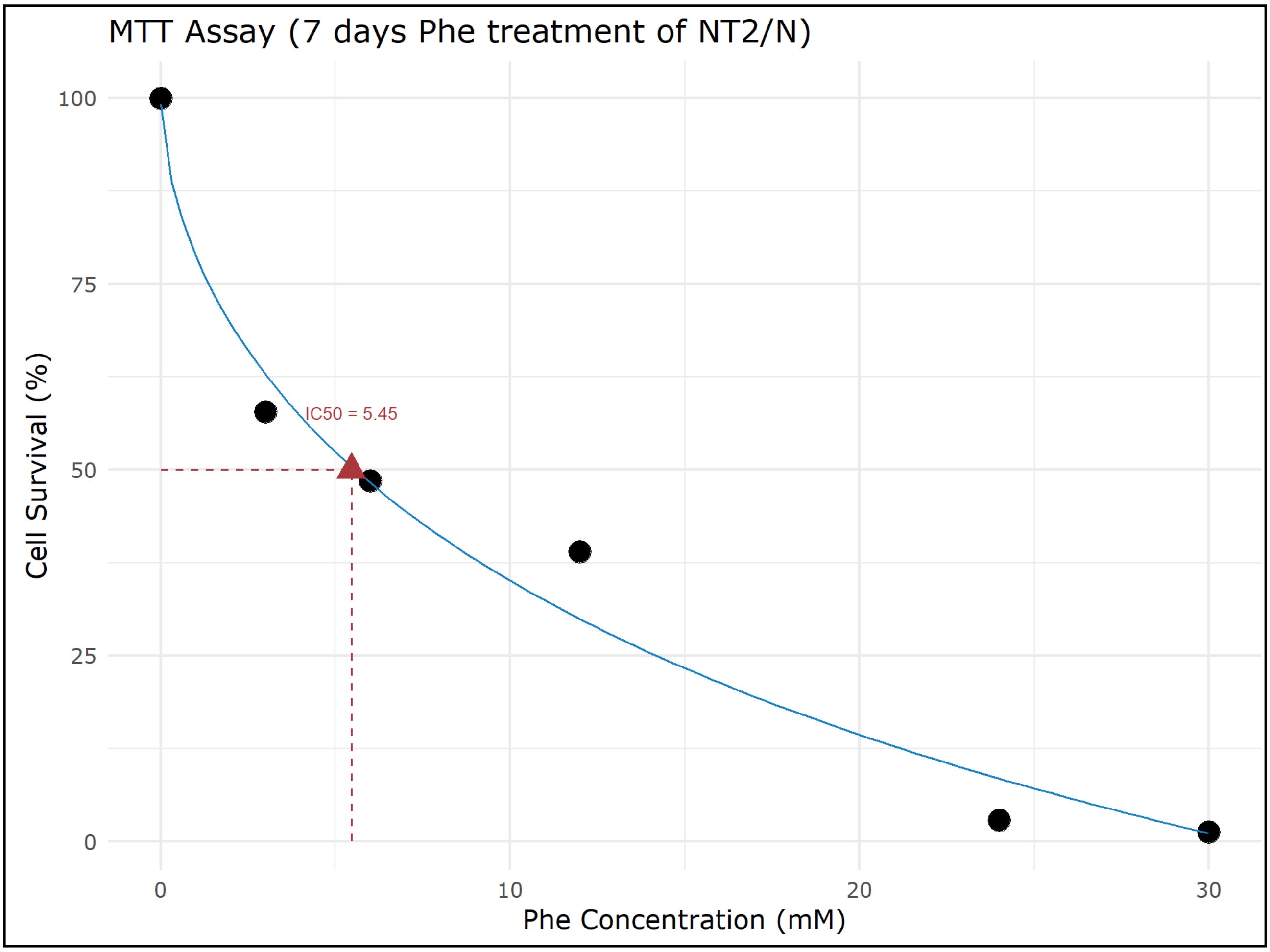

Figure S1. Standard curve of NT2/N cell viability after exposure to Phe. Cell viability was determined using MTT assay after 7 days of NT2/N treatments with 3 mM, 6 mM, 12 mM, 24 mM and 30 mM Phe. After normalization, cell survival was represented as percentage relative to untreated NT2/N cells, which was set at 100%. Standard curve was constructed based on 5 different Phe concentration treatments and untreated NT2/N, and concentration corresponding to 50% cell viability was calculated (5.45 mM).

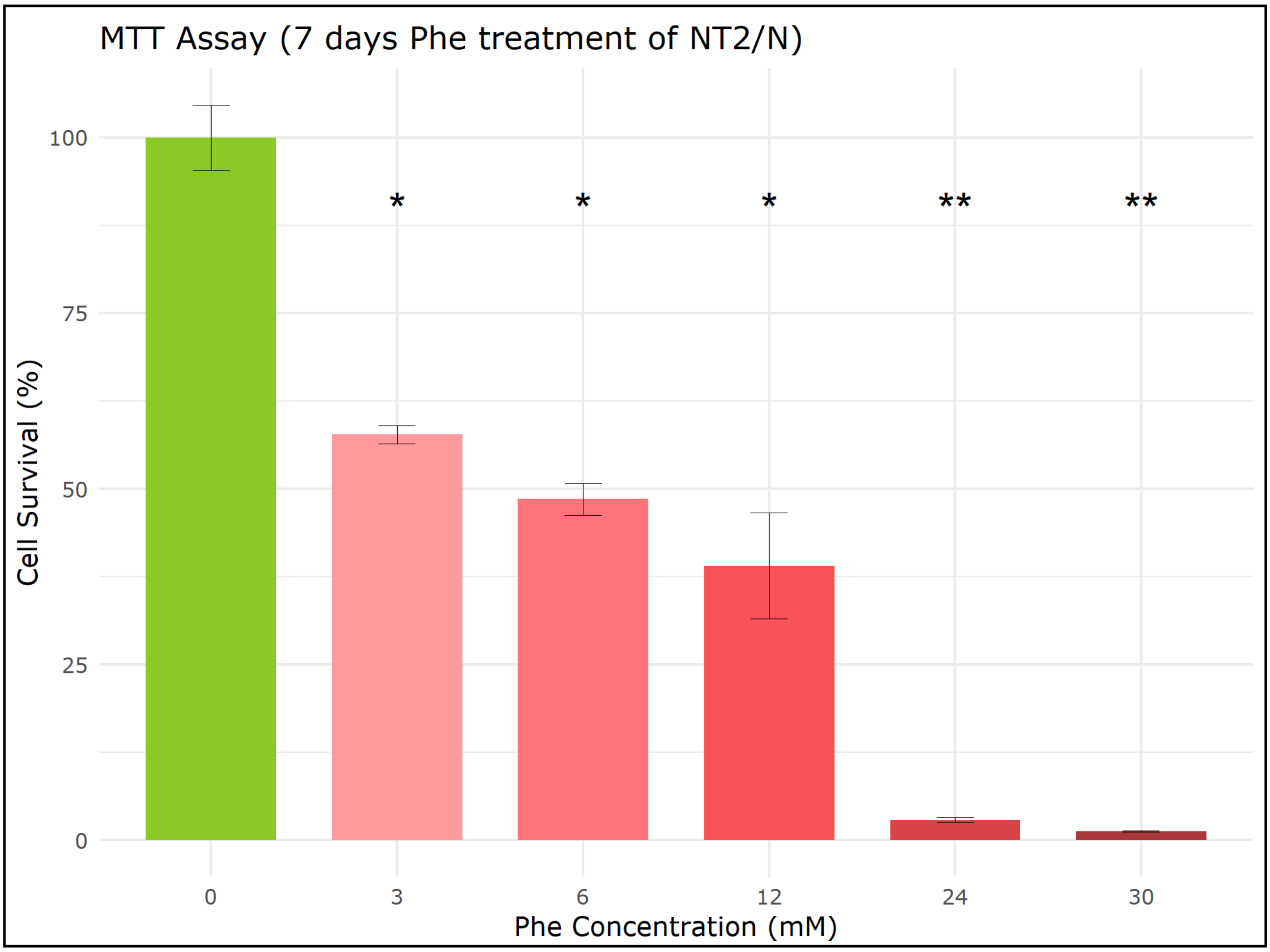

Figure S2. Treatment with increasing concentrations of Phe reduces cell viability of NT2/N. NT2/N were exposed to varying concentrations of Phe (3, 6, 12, 24 and 30 mM) for 7 days. The MTT assay was used to determine cell viability. Absolute values obtained for each Phe concentration group were normalized to the absolute value obtained for untreated NT2/N cells, which was set at 100%. The statistical significance between the treatment groups and untreated NT2/N was estimated by Student's t-test. The error bars represent the means ± standard deviation (SD) obtained from four technical replicates in two independent experimental repeats of the MTT assays. The asterisks indicate a significant difference between groups with * representing p-values between 0.05 and 0.001 and ** representing p-values less than 0.001.

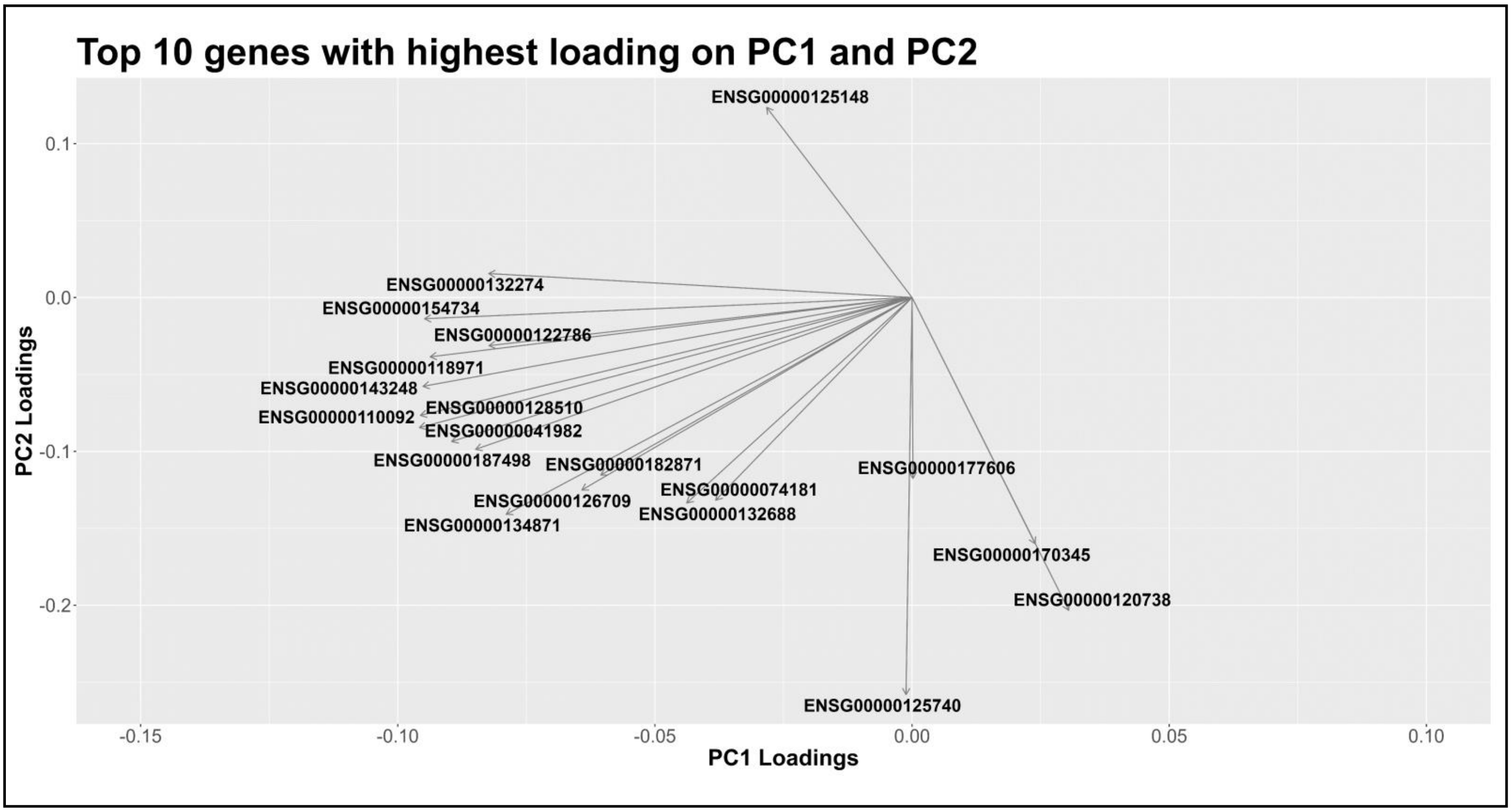

Figure S3. Visualization of top 10 loadings contributing to PC1 and PC2 variation. The gene symbols corresponding to ENSEMBL IDs shown in the figure are: *CCND1* (ENSG00000110092), *CPA4* (ENSG00000128510), *RGS5* (ENSG00000143248), *ADAMTS1* (ENSG00000154734), *CCND2* (ENSG00000118971), *TNC* (ENSG00000041982), *COL4A1* (ENSG00000187498), *TRIM22* (ENSG00000132274), *CALD1* (ENSG00000122786), *FOSB* (ENSG00000125740), *EGR1* (ENSG00000120738), *FOS* (ENSG00000170345), *COL4A2* (ENSG00000134871), *NES* (ENSG00000132688), *NOTCH3* (ENSG00000074181), *IFI6* (ENSG00000126709), *MT2A* (ENSG00000125148), *JUN* (ENSG00000177606) and *COL18A1* (ENSG00000182871).

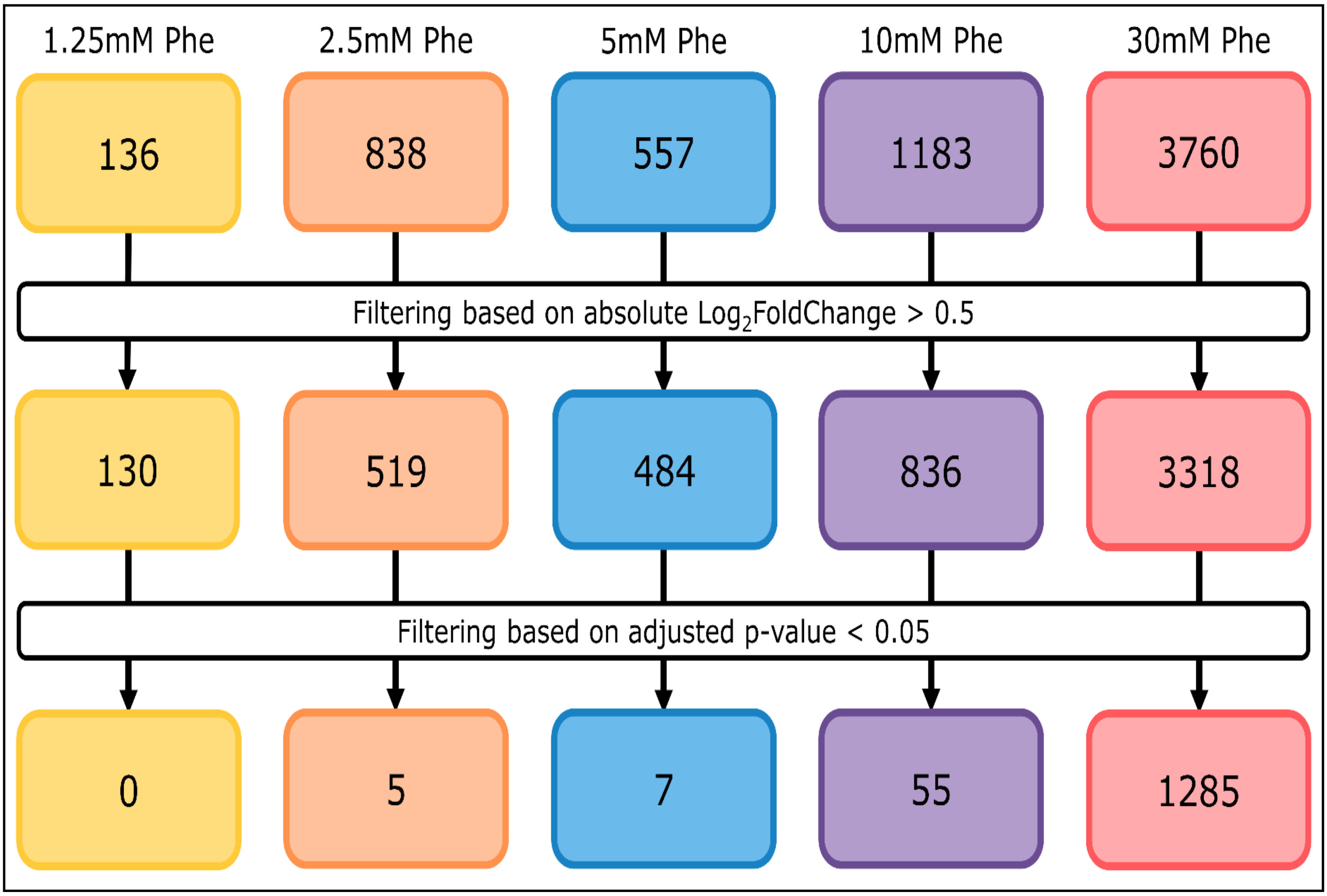

Figure S4. Schematic view of the DEGs analysis and filtering process. After differential expression analysis, the obtained number of DEGs (first row) was filtered based on absolute Log_2FoldChange, decreasing the subset of DEGs. Then, the next filtering was performed based on adjusted p-value (p-value corrected by Benjamini nand Hochberg's method) showing the final statistically significant subset of DEGs for each Phe treatment group.

A

B

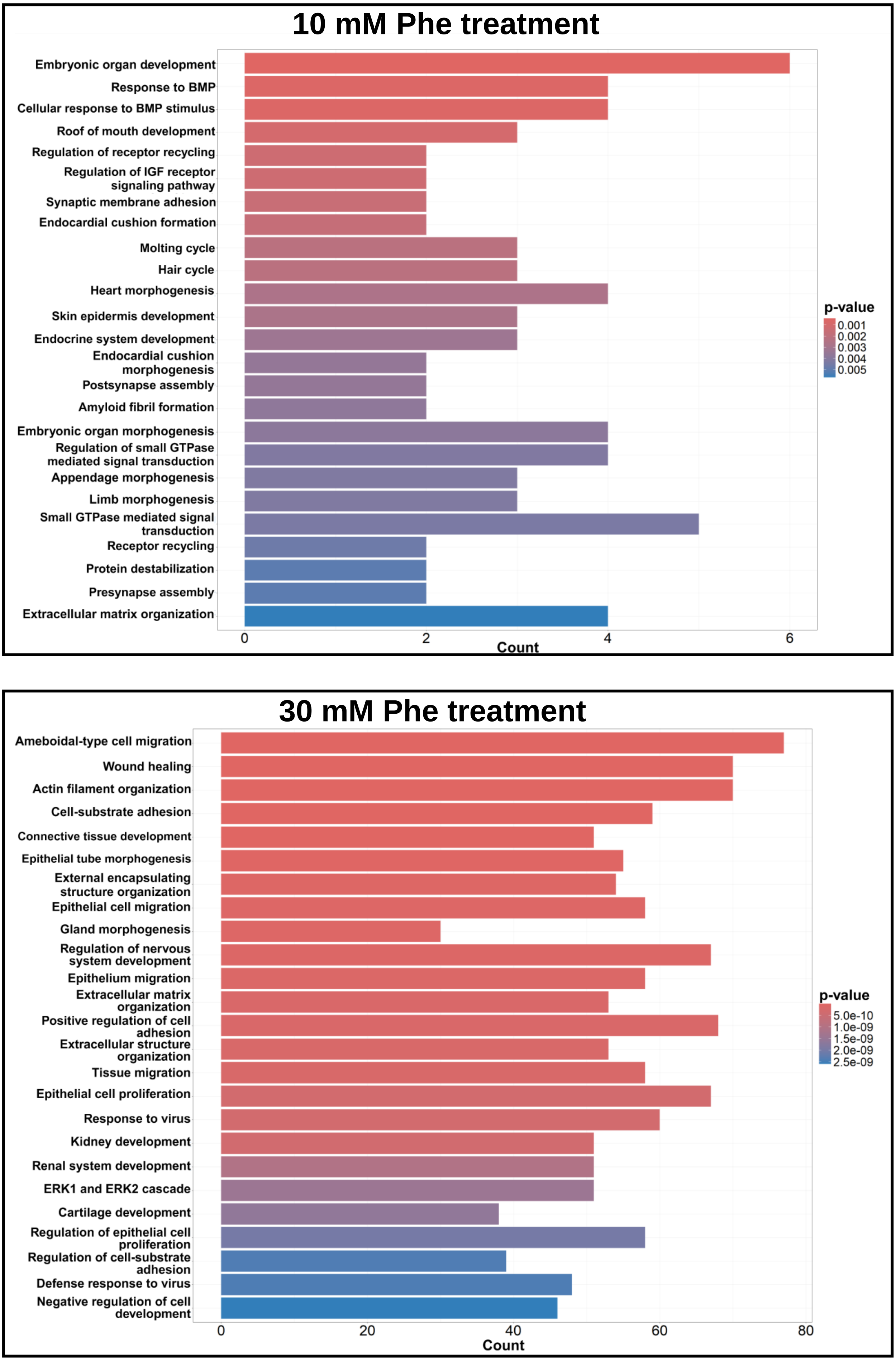

Figure S5. Significant GO terms obtained by overrepresentation analysis of DEGs from 10 mM and 30 mM Phe treatment group. A) Enrichment analysis in 10 mM Phe treatment groups revealed GO terms such as regulation of neuron projection development and arborization, synaptic membrane adhesion and assembly. B) Overrepresented terms upon 30 mM Phe treatment include cell migration, ERK1 and ERK2 cascade, regulation of apoptotic signaling pathway as well as actin filament organization and response to axon injury. Plots represent top 25 overrepresented GO terms.

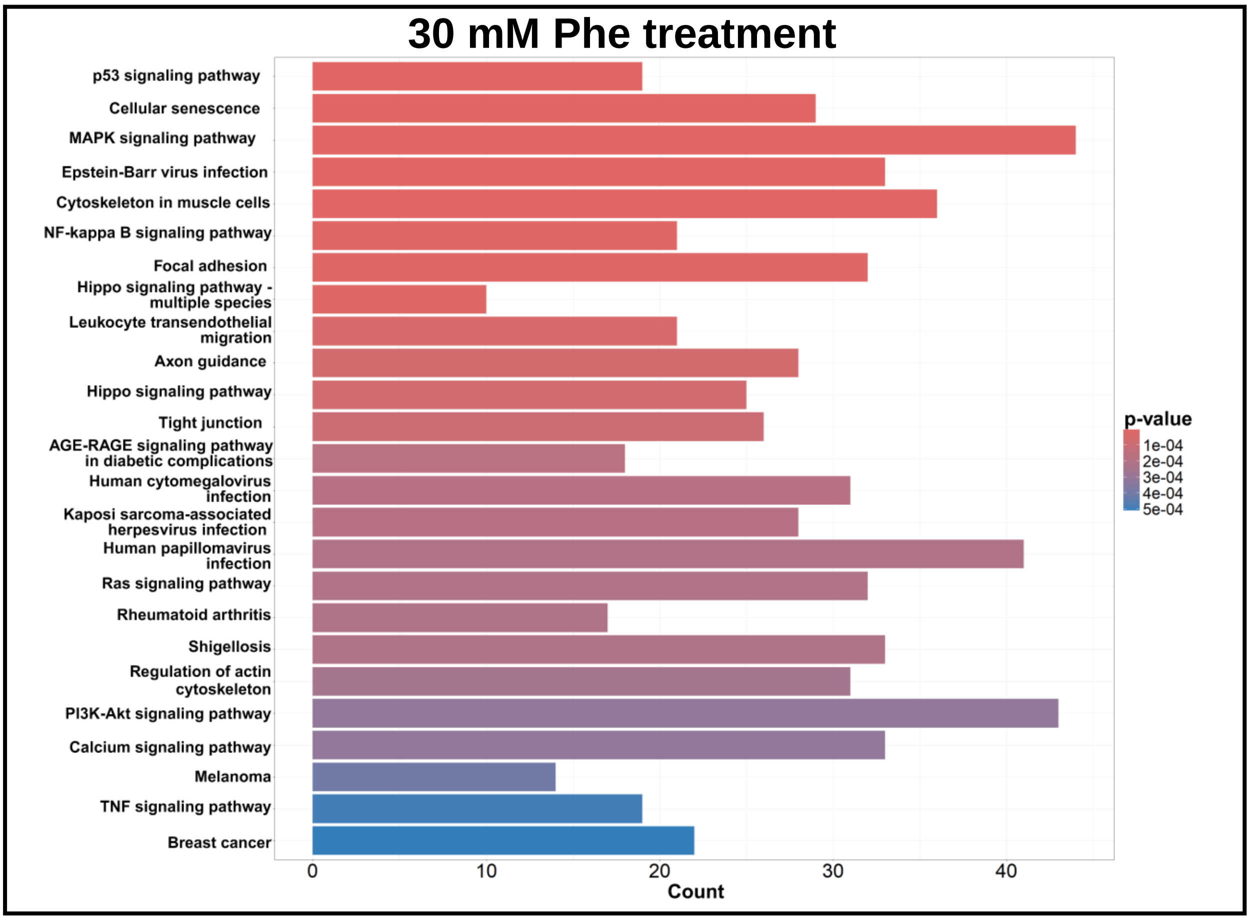

Figure S6. Significantly overrepresented KEGG pathways obtained upon treatment with 30 mM Phe. Among pathways enriched in 30 mM Phe treatment group are the following: cell-substrate adhesion, actin filament organization and ERK1 and ERK2 cascade. The plot represents top 25 enriched KEGG pathways.

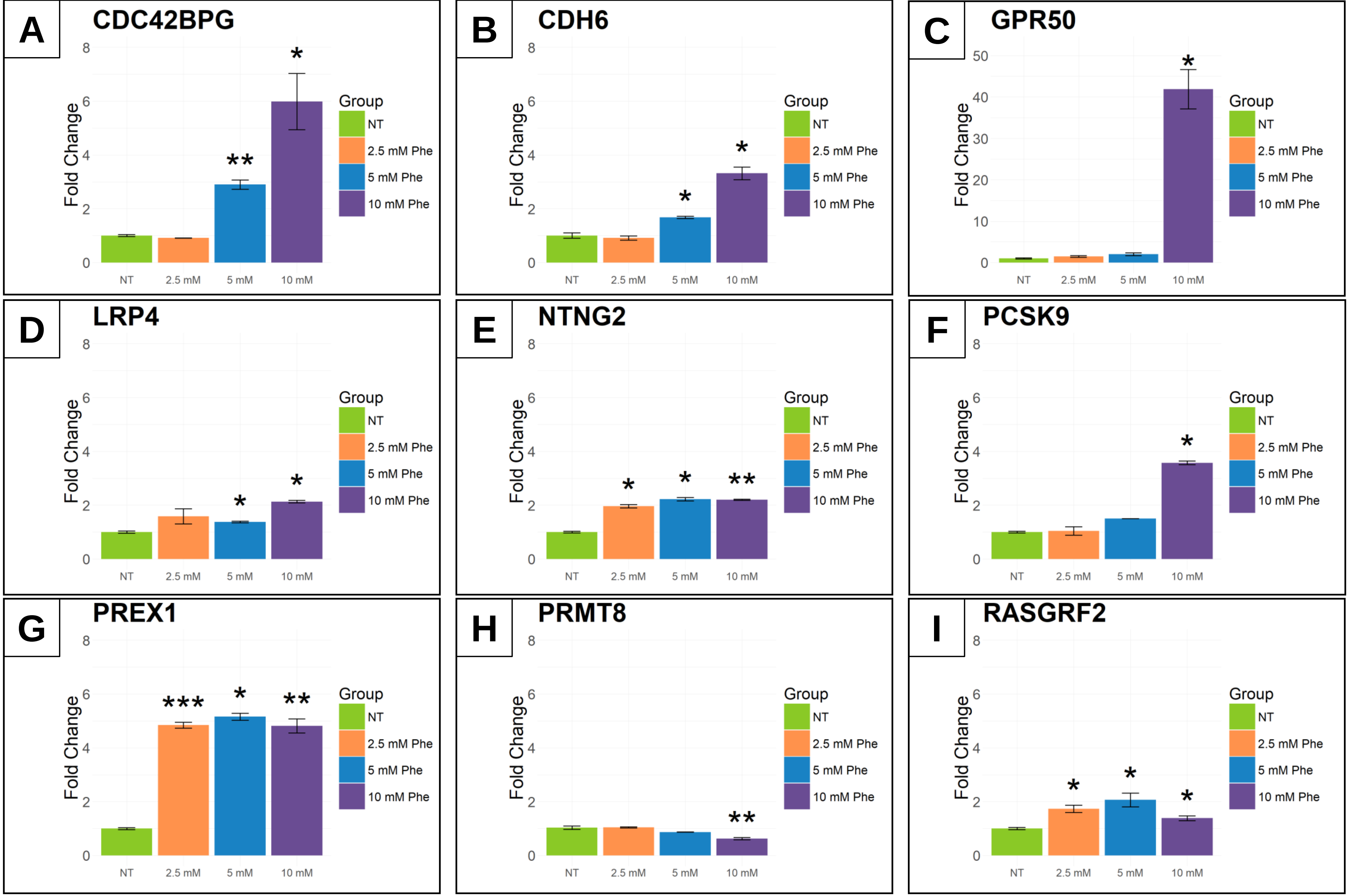

Figure S7. Barplots representing the gene expression changes observed upon qRT-PCR. Gene expression of 9 selected genes was assessed and compared to control cells (NT, green) for samples from 2.5 mM, 5 mM and 10 mM treatment groups. Statistical significance is represented by asterisks on the barplots (*adjusted p-value<0.05, **adjusted p-value<0.01, ***adjusted p-value<0.001, Holm-Bonferroni correction method). Expression of the following genes was assessed by qRT-PCR: A) *CDC42BPG*, B) *CDH6*, C) *GPR50*, D) *LRP4*, E) *NTNG2*, F) *PCSK9*, G) *PREX1*, H) *PRMT8* and I) *RASGRF2*.
